# Ten simple rules to make your publication look better

**DOI:** 10.1371/journal.pcbi.1008938

**Published:** 2021-05-20

**Authors:** Friederike Ehrhart, Chris T. Evelo

**Affiliations:** 1 Department of Bioinformatics—BiGCaT, NUTRIM/MHeNS, Maastricht University, Maastricht, The Netherlands; 2 Department of Bioinformatics—BiGCaT, NUTRIM, Maastricht University, Maastricht, The Netherlands; Dassault Systemes BIOVIA, UNITED STATES

## Introduction

Paying attention to the finishing details of a publication reminds one of pedantic teachers in school days, and students often consider it as waste of time. However, in professional life as a scientist, who lives to create and publish new knowledge, the format of the publication remains important for several reasons.

Poor form of a publication, may it be a scientific paper or a thesis, makes it hard to focus on the content. Readers (including reviewers) stumble upon errors and flaws, get distracted, and even annoyed. Reviewers will spot how much care and attention was paid to the details of the report, and that will affect their expectations about the technical work behind the paper. Thus, simple things like format, spelling, or abbreviation management will affect the understanding and judgment of the paper. From an educational perspective, we want to teach young scientists not only to communicate great ideas but also to pay attention to detail.

Different journals have different preferences and style guides, but there are several items that all of them share. The intention of this Ten Simple Rules paper is to provide general guidelines to produce a scientific publication in good form, using computational biology for examples. Rules 1 to 3 cover layout strategies, 4 to 6 nomenclature and terminology, rule 7 is about supplementary data and code, and rules 8 to 10 deal with error prevention and acceptance. The guidelines should apply for both scientific publications and educational thesis for different levels of students. For good scientific writing, scientific publication practice, or how to display results properly, there are other guides [1–4].

### Rule 1: Decide for one layout and apply that consistently

When a particular format is required, be sure to follow the instructions. Different journals have different preferences when it comes to font, size, margins, alignment, etc. If they offer a draft-formatted document—use it. If a particular format is not required (e.g., *PLOS Computational Biology* does not require a specific format for the first submission), select one (format) and stick to it. The details of the format (font, size) do not matter as much, what matters most is that you stay consistent with the format throughout the document. The same applies for headings—decide once which organization for font, size, block, italics, or underlined style you use, and apply it consistently to the whole document. Using automatic formatting offered by editing programs like Microsoft Word or LaTeX helps to consequently apply that format (see Rule 2).

Consistency is good as long as it is about things that really are the same. If you use quotes or write an intermezzo, it makes sense to use a different style.

### Rule 2: Use automatic formatting tools for headings, figure captions, and references

Text formatting allows one to mark titles, headings, text body, references, or figure captions. It automatically applies the style (font, specific text size, or bold or not) you defined to these text items and allows the creation of automatic lists, e.g., table of content or list of figures. It helps to avoid mixed formatting and really makes work easier once you learn how to handle them.

For reference management programs or add-ons are even more important. You do not want to review 100 references over and over again to check for missing dots, use of bold or italics, and the correct spelling of unusual names. Additionally, you surely do not want to adapt them manually for another journal once you change your mind about where to submit.

### Rule 3: Use correct domain-specific nomenclature

Check the domain-specific nomenclature and apply it consequently. Domain-specific nomenclature may imply the use of SI units for units of measurement, the recommended international nonproprietary name for drugs, etc. In the computational biology domain, the nomenclature for genes, genetic variants, and species are among the most important ones, so these are provided here as examples:

(1) Genes, transcripts, and proteins: Most journals today prefer the use of gene symbols defined and given by the Human Genome Organization (HUGO) Gene Nomenclature Committee (HGNC; https://www.genenames.org/about/guidelines). The nomenclature allows differentiation between gene, transcript, and protein as well as differentiation between human and animal genes. If what you are referring to is a specific protein product or a specific transcript, then you will often want to refer to a specific database entry in, for instance, UniProt or ENSEMBL. In that case, it is best to use the name used by that database in combination with a compact identifier or that database entry.

(2) Genetic variants: There is more than one way to correctly identify a genetic variant. One common way utilizes identifiers from dbSNP with reference SNPs (e.g., rs1234567). Another more human-readable nomenclature is defined by the Human Genome Variation Society (HGVS) (https://www.hgvs.org/; http://varnomen.hgvs.org/). Independent, which you choose, make sure to stick to the chosen nomenclature to correctly identify your variants.

(3) Species: Since Carl von Linné, the species name is defined as a combination of genus name and species name. Species names are written in *italics*. Humans—*Homo sapiens*—belong to the genus *Homo*, and our species name is *Homo sapiens*. Note that the genus name is capitalized, but the species name is not. Species names may be abbreviated by reducing the genus name to the capital like this: *H*. *sapiens*.

### Rule 4: Manage your abbreviations

Typically, any technical text becomes less readable if abbreviations are used that are not intuitively understood or already known by the reader. If the main reason to introduce abbreviations is that you do not want to type the same long words over and over again, you can consider using an abbreviation during the writing process that you replace with the full text at the end. If you use an abbreviation only once or twice in the text, you really do not need it. *PLOS Computational Biology*, e.g., does not recommend use of “non-standard abbreviations unless they appear at least three times in the text” (https://journals.plos.org/ploscompbiol/s/submission-guidelines#loc-abbreviations). Use as many abbreviations as necessary, but as few as possible.

Introduce an abbreviation by writing the full expression followed by the abbreviation in brackets and introduce the abbreviation in both the abstract and main text body because abstract and full text are considered independent text entities. They can be published in different places and must be readable when separated from each other.

Repetition of an abbreviation may be useful at the beginning of a new section. Keep in mind that readers might read your paper in a different order than you wrote it, and especially, things like figure captions (see, e.g., “Writing an Effective Figure Legend” by M. Panter https://www.aje.com/en/arc/writing-effective-figure-legend/) and table headers should be readable by themselves. Gene symbols are not abbreviations and may be used without explanation. There are a few abbreviations that are so common (in their respective domain) that you do not need to introduce them, e.g., DNA, RNA, *et al*., or PCR.

Stick to one abbreviation per term, and once introduced, use it consequently. It helps to make a list of abbreviations while writing to keep an overview, although not all journals want a separate list of abbreviations submitted with the text.

### Rule 5: Use the correct form of Latin expressions and abbreviations

Latin expressions and their abbreviations are traditionally written in italics, although some of them became so common that they are integrated in English language by now and not all writing guides require use of italics any more. The most commonly used Latin abbreviation is *et alii* (and others), whereas the correct abbreviation is *et al*. (note the single dot). Common examples are *in vivo*, *in vitro*, and *in situ* for experimental descriptions, *vice versa* (the opposite of what was said is also applicable), and *e*.*g*. (*exempli gratia*—for example) and *i*.*e*. (*id est*—in other words) which are frequently mixed up. Less often seen but still used is *i*.*a*. *(inter alia)* which translates to “among other(s)”.

### Rule 6: Use non-breaking spaces

Non-breaking space (also referred as “no-break space” or “hard space”) is a special space character that does not allow a line break in that exact position. These are used to prevent a new line starting with something strange. The most common and only obligatory use in scientific literature is between numbers and their units (e.g., 10_μg), but non-breaking spaces can also be used between days and months (e.g., June_3rd), within a name (e.g., Jon_Doe, Elizabeth_II), or in any other situations in which breaking lines may be disruptive for the reader (e.g., in an infobox). Different text editing programs have different shortcuts to add those, e.g., in Microsoft Word, it is Ctrl-Shift-space, and in LaTeX, you can use “~”.

### Rule 7: Pay attention to the supplementary data and code

Supplementary data and code are often very valuable as they allow reproduction and reuse of the materials described in the paper, and several data types like genetic sequences or omics data are mandatory to deposit and publish together with the paper. Make sure the link is persistent, and the data are actually there. The proper way to store raw or processed data or cite data (data citation according to DataCite standard) is described elsewhere [5–7].

For software, including code that is used to generate figures, software packages, and workflows, there are specialized repositories that allow version control and guarantee accessibility and online availability (e.g., GitHub and Bioconductor). See also reference [8] for a more detailed description of how to document or to cite [9] scientific software properly.

Supplementary tables are often given as Microsoft Excel formats. The automatic recognition of gene symbols in Excel and transfer to a date format, e.g., SEPT7 becomes September 7^th^, was a common problem; it occurred in up to 20% of supplementary data Excel files in the past [10]. However, this has been resolved by the latest update of the HGNC symbols. For example, SEPT7 is now SEPTIM7 and no longer triggers Excel’s auto format function.

Make sure to reference any supplementary data or software appropriately in the text, whether in an external repository or supplementary table file, and link the identifier from the respective repository (e.g., Gene Expression Omnibus and ArrayExpress).

### Rule 8: Do a spelling and grammar check

This is the simplest rule and possibly the one most often disregarded. Every advanced editing program has a built-in spelling and grammar check, which should be used at least once before submitting to find the most obvious and most embarrassing errors. Check also whether your own (language specific) automatic spelling correction mechanism did not change anything into more common words, especially when it comes to gene names and symbols (see Rule 7).

Then, there are the errors that the built-in spelling and grammar check will not find (yet). For example, differentiating between “a treating physician” and “a threatening physician” cannot currently be performed automatically, but only by careful proofreading.

In addition to proofreading, make sure to write in the correct tense. We recommend the present tense for the Introduction because it is a description of the present state of the art. Materials and methods and Results are usually written in the past tense as these are things you have performed and have found in the past. Try to minimize passive language for better readability. Good spelling and grammar checking is, however, not a replacement for well-written English. If none of the authors are fluent in English, it remains a good idea to have someone check it who is.

### Rule 9: Review the document again after creating the final version

Before creating a pdf for submission or for printing, make sure that all track changes and comments are resolved and removed from the text. During the creation of a pdf, weird things may happen to your text, so it is important to review the product again at least one more time. Text blocks and items like figures, tables, or headings may have moved. Make sure that headings are not left at the bottom of a page when they should be at the top of the next page. If you have figures or tables in the text, check that they are not separated from their captions or headings. In contrast to graduation thesis, journals usually require the figures to be submitted separately and the tables to be added at the end. Check if specifically formatted text or inserted items (e.g., formulas, special characters, or references) are still shown correctly, and check if hyperlinks to internal (headings) or external (websites) sources work.

### Rule 10: Accept that there will be errors

The aforementioned nine simple rules should help to catch most of the errors and minimize mistakes for a more professional presentation of your work. Despite all effort and proofreading it may, and at some point will, happen that you find an error in your published work. Forgive yourself; absolute perfection is unrealistic. Remember the perfect can even be the enemy of the good—at some stage, you have a valuable manuscript that you want to be published without further delay.

If the error is of such a type that you really, really want to remove it from the publication, the first thing to do is talk to and reach an agreement with the coauthors so they support the decision, and then contact the editor of the journal. Together with the editor, you decide if a new version of record needs to be created. For this, you will have to write a corrigendum or an erratum, which will accompany the paper forever.
